# Comparison of two different doses of dexmedetomidine in attenuating cardiovascular responses during laryngoscopy and endotracheal intubation: A double blind, randomized, clinical trial study

**Published:** 2015

**Authors:** H Jarineshin, A Abdolahzade Baghaei, F Fekrat, A Kargar, N Abdi, S Navabipour, A Zare, H Akhlaghi

**Affiliations:** * Anesthesiology, Critical Care and Pain Management Research Center, Hormozgan University of Medical Sciences, Bandar Abbas, Iran; ** Student Research Committee, Jahrom University of Medical Sciences, Jahrom, Iran; *** Department of Community Medicine, Hormozgan University of Medical Sciences, Bandar Abbas, Iran; **** Student Research Committee, Hormozgan University of Medical Sciences, Bandar Abbas, Iran

**Keywords:** dexmedetomidine, hemodynamic, intubation, endotracheal

## Abstract

**Introduction.** Secure airway for proper ventilation during anesthesia is one important component of a successful surgery. Endotracheal intubation is one of the most important methods in this context. Intubation method and used medication are considerably important in attenuating complications. This research aimed to investigate the impact of two different doses of dexmedetomidine in mitigating cardiovascular responses to endotracheal intubation in candidate cases supporting voluntary operation.

**Methods. ** The current research contained 90 cases in the range of 18 and 50 old, with ASA I,II supporting voluntary operation, who were randomly classified into three teams, each group consisting of 30 cases. The first set (A) got 0.5 μg/ kg dexmedetomidine, the second set (B) got 1 μg/ kg dexmedetomidine and the third set (C) got an equal volume of saline as placebo, 600 seconds earlier the initiation of anesthesia. Hemodynamic parameters were recorded at baseline (T0), then after the injection and the earlier initiation of anesthesia (T1), after the induction of anesthesia and before the endotracheal intubation (T2), promptly after tracheal intubation, 180, and 300 after endotracheal intubation (T4, T5). Data was analyzed and p < 0.05 was supposed notable.

**Findings.** In this research, 3 teams were similar regarding weight, age, height, sex and duration of laryngoscopy. The diastolic mean arterial pressure, heart rate, and systolic arterial pressure were significantly lower in dexmedetomidine teams (A,B) at all times after the endotracheal intubation compared to group C. There were no significant differences in hemodynamic factors among group A, B.

**Conclusion. ** Dexmedetomidine effectively and significantly attenuates cardiovascular and hemodynamic responses during endotracheal intubation. In addition, different doses of dexmedetomidine did not cause any significant distinct result in mitigating cardiovascular responses.

## Introduction

The anesthesiologist is mainly responsible for providing a secure airway for a proper ventilation of the patient during anesthesia and surgery [**1**]. No medication and anesthetic method is reassuring, unless a secure airway is maintained with great efforts. Laryngoscopy and endotracheal intubation is a commonly used measure for the maintenance of a secure airway during general anesthesia and it has specific indications [**2**].

Endotracheal intubation leads to a painful stimulus, which causes severe physiological responses such as autonomic and activated brain stem reflexes [**3**]. Direct laryngoscopy and endotracheal intubation directly affect severe sympathoadrenal responses, which increase arterial blood pressure, plasma catecholamine levels, heart rate, and even lead to dysrhythmia in some cases [**2**]. Usually, the vascular contraction reflex is manifested in a few seconds and sinus tachycardia culminates during the first two minutes and lasts for five minutes. These changes can be threatening and risky for hazardous patients with high blood pressure, coronary artery disease or high intracranial pressure [**2**,**4**]. Various methods and medications are used to control the hemodynamic responses to laryngoscopy and endotracheal intubation such as advancing anesthesia depth, minimizing duration of intubation (less than 15 seconds), administrating drugs such as intravenous and endotracheal lidocaine, short-acting opioids, beta-adrenergic blockers, calcium channel blockers, vasodilator drugs and even magnesium [**3**,**4**].

A selective drug and medication depend on the duration of surgery, urgency of the surgery, anesthetic technique, and routes of drug administration, patient medical conditions, and patient willingness to anesthetic procedure. Dexmedetomidine is an alpha-2 adrenergic receptor agonist, which specifically binds to alpha-2 receptor [**5**]. Adrenergic alpha-2 agonist reduces heart rate and blood pressure [**6**]. Dexmedetomidine demonstrates sedative and analgesic impacts, and it is utilized for intravenous sedation in the intense care section [**7**]. Sedative impacts of this drug are induced through the stimulation of alpha-2 adrenoceptor. As a result, dexmedetomidine is commonly used prior to surgery [**8**]. Alpha-2 agonists are dexmedetomidine and clonidine, which reduce sympathetic outflow and decrease cardiovascular behavior to operational and laparoscopic stimuli during surgery [**9**]. These drugs decrease tachycardia, hypertension, and sympathetic activity, which are beneficial for the cases with a presence of myocardial ischemia [**10**]. Although several studies have confirmed beneficial effects of these drugs, results of some studies showed no relationship between these drugs and decreased cardiovascular complications during tracheal intubation.

**Objective of the study**

The present study aimed to investigate the impact of two various doses of dexmedetomidine in attenuating cardiovascular responses to tracheal intubation in candidate cases supporting voluntary operation, in Shahid Mohammadi Hospital in Bandar Abbas during 2013 and 2014.

**Sampling Method**

This was a double-blind prospective clinical case. The sample size was calculated according to literature. The information obtained from the study executed by Smith [**11**] was used to evaluate the instance model size in the investigation of variance.

α = 0.05, β = 0.2, σ = √MSE = 12/ 11,

μ1 = 76.3, μ2 = 71.9, μ3 = 91.03

**Figure:**
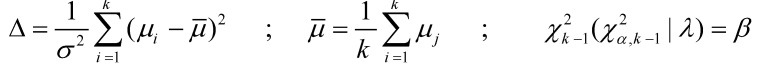


The number in each group was the following:

**Figure:**
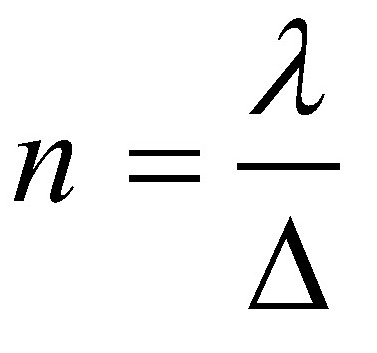


λ value was equal to 9.64 according to α = 0.05, β = 0.2 and decentralized chi-square distribution. The sample size was estimated at 27 people in each group by calculating Δ (the sample size was calculated by using NCSS software). The patients were randomly divided into three groups according to the table obtained from the Random Allocation Software.

**Inclusion Criteria**

In this study, 90 patients in the range of 18-50 years old, with ASA I and II (based the on the distribution of Anesthesiologists American Society), who referred to Shahid Mohammadi Hospital of Bandar Abbas for elective surgery between 2013 and 2014, were selected.

**Exclusion Criteria**

Patients with ASA > 2, cases with a history of cerebrovascular problems or a history of seizures, cases with full stomach and emergency surgery, opium addicts, cases with a drug allergy history, asthmatic cases, and those treated with drugs such as calcium channel blockers, beta-blockers and ACE inhibitors, were excluded from the study. Patients with prolonged endotracheal intubation (over 30 seconds) or the patients with failure in the endotracheal intubation at the first attempt or the patients experiencing complicated laryngoscopy and intubation processes, were also obtained from the research.

## Materials and Methods

**Management of Anesthesia**

Knowledgeable assent was taken from all cases for the research. A code of ethics was also obtained from Hormozgan University of Medical Sciences (3-hec- November 11, 2014).

Both male and female patients were involved in the investigation. All the cases got 5-7 ml/ kg of balanced salt solution after obtaining intravenous line and before the induction of anesthesia. Regular controlling devices comprising pulse oximetry, noninvasive blood pressure (NIBP) cuff, electrocardiography (ECG), capnography (S/ 5 anesthetic monitor [Datex-Ohmeda, Finland]) were used for monitoring. Baseline vital signs of all patients were measured and recorded such as systolic blood pressure (SBP), heart rate (HR), diastolic blood pressure (DBP), mean arterial pressure (MAP) and arterial blood oxygen saturation percent (SPO2). The patients were enrolled into the following groups based on computer program of table of random allocation: 1) The first group of patients received solution A; 2) the second team of cases received solution B; 3) the third team of cases received solution C. The solutions were daily prepared in 10-cc syringes labeled as A, B and C by an anesthesiologist. For this purpose, 0.5 μg/ kg dexmedetomidine was used to prepare Solution A; 1μg/ kg dexmedetomidine was used to prepare Solution B; an equal volume of normal saline was used to prepare Solution C. All the solutions were prepared in 10ml volume and were administered by an anesthesiologist 600 seconds earlier to the induction of anesthesia. Then, all the cases received fentanyl (2.5 μg/ kg) and intravenous midazolam (0.05 mg/ kg) as premedication. Induction of anesthesia was similarly done for all patients with propofol (1-1.5 mg/ kg) and cisatracurium (0.2mg/ kg). After an adequate muscle relaxation (TOF=0), tracheal intubation was conducted by an experienced anesthesiologist. Hemodynamic parameters were measured and recorded at the following periods in addition to baseline (T0).

T1: after injection of medication and before the induction of anesthesia

T2: After anesthesia induction and before the intubation of endotracheal tube 

T3: promptly after the intubation of endotracheal tube 

T4: 180 seconds after the intubation of endotracheal tube

T5: 300 seconds after the intubation of endotracheal tube 

Demographic data were recorded in a special form. This form had two parts. The first part was relevant to information regarding the patient (file number, name, and surname, age, gender, height, weight). The second part was relevant to specialized data of the project (type of surgery, a history of underlying disease, studied group, a history of drug use, SBP, DBP, MAP, HR, SPO2, arrhythmia incidence, duration of laryngoscopy, the number of laryngoscopy and intubation procedures and possible complications).

**Statistical Analysis**

The data were entered into SPSS version 16. Kolmogorov-Smirnov and chi-square statistical tests were used to examine the normal distribution of the studied variables. Renewed Measures of Variance Investigation and Friedman non-parametric test were used to find the difference among the three teams at different times of T0 to T5. Wilks’s Lambda Test was used to check the effect of time and time-group interaction with the studied parameters. Sphericity Assumed Test was used to analyze the collected data at different times. Given that the three groups were included in this study, Bonferroni Post Hoc Test was used to reveal the distinctions observed in factors among the teams. Kruskal Wallis One-Way Variance Investigation was used to compare the studied parameters in the three groups each time. The significance level was considered as p < 0.05.

**Findings**

The demographic characteristics and duration of laryngoscopy were identical in the three teams and had no significant distinction (**Table 1**).

**Table 1:** Demographic characteristics and duration of laryngoscopy of the patients in the three groups

	Group A	Group B	Group C	
	0.5 µg/ kg Dexmedetomidine	1 µg/ kg Dexmedetomidine	Control	p-value
Age	29.33 ± 8.01	29.67 ± 9.07	29.53 ± 7.42	0.917
Sex (male/ female)	20/ 10	19/ 11	19/ 11	0.953
Weight (kg)	65.37 ± 9.07	64.73 ± 8.79	64.67 ± 7.87	0.932
Height (cm)	169.10 ± 7.08	169.40 ± 6.60	168.73 ± 7.00	0.935
Duration of laryngoscopy (second)	20.3 ± 3.68	20.1 ± 2.97	19.3 ± 3.97	0.495

The comparison of SBP between the groups showed that the mean systolic blood pressure from T2 to T5 were clearly higher in team C than in team A, B (p < 0.05).

**Fig. 1:**
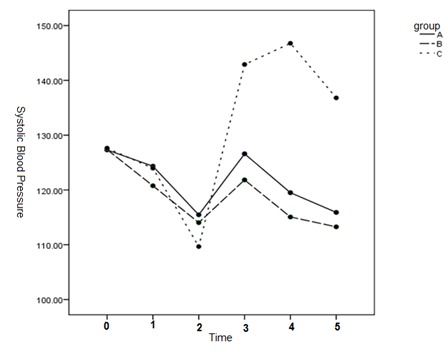
Mean SBP at different times between the 3 groups

No statistically meaningful distinction was seen in the mean SBP among teams A and B at T2, T3 and T5 (p > 0.05) (p = 0.292) but group A had a statistically significantly higher mean SBP than group B at T4 (p < 0.05) (**Table 2** and **Fig. 1**).

**Table 2:** The mean ± Standard deviation of the parameter in this study at different time periods

		Group A	Group B	Group C	
	Time	Dexmedetomidine 0.5 µg/ kg	Dexmedetomidine 1 µg/ kg	Control	P value
SBP	T0	127.3 ± 8.7	127.3 ± 8.6	127.6 ± 8.6	0.988*
	T1	124.3 ± 6.7	120.7 ± 7.1	123.9 ± 8.7	0.141
	T2	115.4 ± 6.6	114.0 ± 6.7	109.6 ± 6.9	0.004
	T3	126.6 ± 6.0	121.8 ± 7.3	142.9 ± 11.3	<0.001
	T4	119.5 ± 5.2	115.0 ± 6.8	146.7 ± 7.4	<0.001
	T5	15.9 ± 4.7	113.2 ± 5.7	136.8 ± 6.5	<0.001
DBP	T0	74.3 ± 6.6	74.6 ± 6.7	73.5 ± 6.7	0.795
	T1	72.0 ± 5.8	71.0 ± 6.1	71.9±6.4	0.780
	T2	67.5 ± 5.8	67.1 ± 6.3	65.3±5.4	0.298
	T3	73.0 ± 6.0	67.8 ± 5.6	81.5±5.0	<0.001
	T4	68.4 ± 6.2	67.8 ± 5.6	81.5 ± 5.0	<0.001
	T5	66.5 ± 5.5	66.5 ± 5.9	77.2 ± 4.2	<0.001
MAP	T0	88.5 ± 7.9	89.4 ± 6.9	89.9 ± 7.3	0.762
	T1	86.4 ± 5.7	84.7 ± 5.8	88.0 ± 6.9	0.133
	T2	80.4 ± 6.0	79.2 ± 5.9	79.0 ± 5.6	0.625
	T3	87.5 ± 5.9	84.2 ± 6.3	99.7 ± 7.7	<0.001
	T4	82.0 ± 5.2	79.5 ± 5.5	101.8 ± 5.4	<0.001
	T5	79.3 ± 5.7	78.0 ± 5.3	96.0 ± 4.7	<0.001
HR	T0	81.4 ± 11.0	79.8 ± 10.2	80.7 ± 10.2	0.840
	T1	73.6 ± 8.7	71.1 ± 8.1	77.6 ± 10.3	0.024
	T2	69.0 ± 8.7	65.5 ± 7.3	77.5 ± 9.4	<0.001
	T3	76.3 ±10.6	71.9 ± 8.5	93.7 ± 8.1	<0.001
	T4	70.9 ± 9.2	66.1 ± 7.1	92.2 ± 7.8	<0.001
	T5	68.6 ± 8.5	65.2 ± 6.9	86.9 ± 7.4	<0.001
*p < 0.05 is significant					

**Fig. 2:**
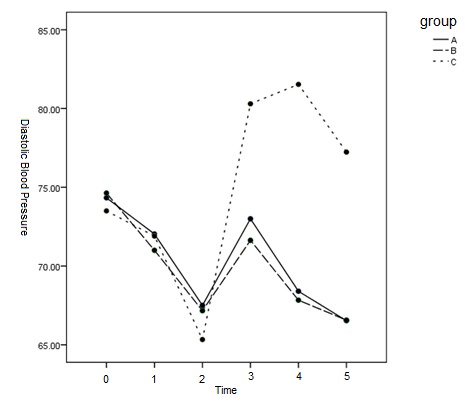
Mean DBP pressure at different times between the 3 groups

The results of comparison of DBP showed that the mean DBP was significantly higher in group C from T3 to T5 than in groups A and B (p < 0.05). No statistically significant difference was observed in the mean DBP between groups A and B (p = 1.000) (**Table 2** and **Fig. 2**).

**Fig. 3:**
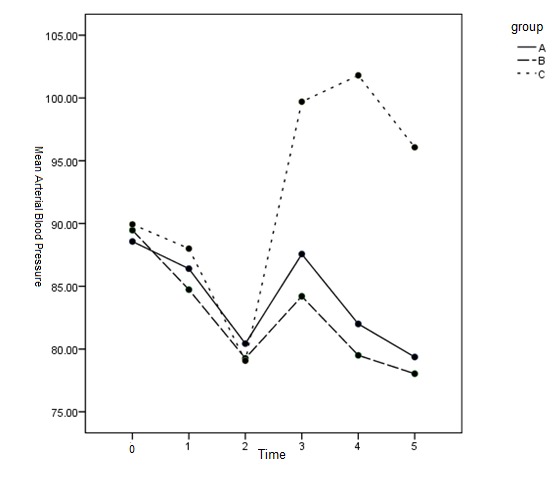
MAP pressure at different times between the 3 groups

The results of comparison of MAP showed that MAP pressure was clearly higher in team C from T3 to T5 than in groups A and B (p < 0.001>). No statistically clear variation was seen in MAP pressure among teams A, B (p = 0.879) (**Table 2** and **Fig. 3**).

**Fig. 4:**
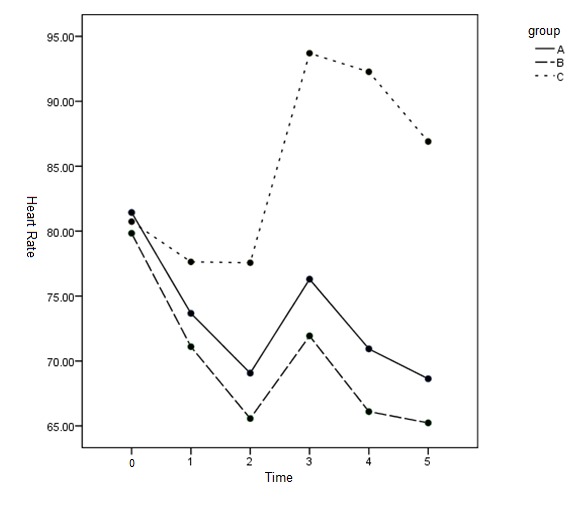
The mean HR at different times between the 3 groups

Results of comparison of mean HR showed that the mean HR in team C was significantly distinct from team B at T1 (p < 0.019). In addition, the mean HR was significantly more in team C than in teams A, B from T2 to T5 (p < 0.001). No statistically clear variation was seen in mean HR among teams A, B (p = 0.327) (**Table 2** and **Fig. 4**).

## Discussion

Anesthesia induction, laryngoscopy, and intubation of endotracheal tube are linked with the hemodynamic variations and sympathetic system activity that should be considered in high-risk patients [**12**]. Intubation and laryngoscopy are associated with an increased heart rate, blood pressure, and cardiac arrhythmias [**13**]. Many factors affect cardiovascular responses during endotracheal intubation and laryngoscopy. These factors are age, used medications, duration of intubation, depth of anesthesia, etc. The duration of laryngoscopy is the most important factor, which affects cardiovascular responses [**14**]. In this study, the patients who had a prolonged intubation (> 30 seconds) or the patients experiencing a complicated intubation were excluded from the study.

Heart rate declines with age. These changes are more highlighted in young people. In this study, 18 to 50 years old patients were selected to better examine these changes [**15**,**16**]. 

Blood pressure increasing typically five seconds after laryngoscopy, reaches the maximum rate within 1-2 minutes and lasts for 5 minutes [**17**]. Many techniques were used for attenuating the cardiovascular responses but none of them led to favorable responses. Such methods such as increasing the depth of anesthesia, high doses of opiates (which suppresses the hemodynamic response but increases the risk of respiratory depression), aerosols and other local anesthetics were also used, which were associated with milder cardiovascular responses [**18**-**20**]. The medications that were primarily effective on the cardiovascular system, which could decrease HR or BP, were also studied. However, these medications usually did not simultaneously affect the two parameters mentioned earlier. These medications can cause hypotension or bradycardia. Labetalol and esmolol in combination with opioids were also used to decrease cardiovascular complications [**21**,**22**]. 

Identifying benefits of alpha-2 agonists can reduce the need for other anesthetics in the patients receiving clonidine for a long term [**23**]. Agonist impacts of dexmedetomidine are manifested though stimulation of alpha-2 receptors in locus coeruleus. Hypnotic effects are manifested through influencing the same receptors in locus coeruleus and analgesic effects are manifested through influencing spinal cord [**7**,**24**]. Dexmedetomidine selectively stimulates the alpha-2 receptor. The selectivity rate for dexmedetomidine is 1620: 1 compared to its counterpart (clonidine) with a selectivity ratio of 220: 1 for the receptor [**25**]. Thereby, dexmedetomidine decreases the mean heart rate and systemic vascular resistance. Dexmedetomidine causes the mild to moderate the decreases in the tidal volume and the slight changes in respiratory rate [**26**]. Dexmedetomidine has a 6-minute half-life with a rapid distribution phase. The half-life elimination of dexmedetomidine lasts for 2 hours [**27**]. 

SBP was decreased in the three groups from T0 to T2 and was increased after T2. This increase in blood pressure was significantly lower in teams A and B than in team C. SBP in groups A and B, who received dexmedetomidine, did not even reach the baseline value after T2 but SBP was clearly increased in team C after T2. In addition, SBP was significantly less in team B than in A in T4. 

DBP and MAP were significantly higher in group C than in groups A and B after T3. DBP and MAP in groups A and B receiving dexmedetomidine were lower than T0 at all times. There was no meaningful distinction between the two teams.

Smitha et al. [**11**] showed that 1μg/ kg of dexmedetomidine significantly decreased all parameters such as SBP, DBP, MAP compared to the other two teams. A notable variation was seen between the group receiving 1μg/ kg dexmedetomidine and the other two teams. This clear distinction was not seen in our study. In this study, two dexmedetomidine doses (0.5 μg/ kg and 1 μg/ kg) decreased SBP, DBP, and MAP compared to the control team. An important difference between our research and the research conducted by Smitha [**11**] is maybe related to the mean patient ages. In the former study, the mean age was 29 years old while the mean age in the latter study was between 39 and 42 years old. Girish et al. [**28**] showed that MAP and HR were clearly reduced in the patients receiving dexmedetomidine, also present in our findings. Ferdi Menda et al. [**29**] showed that SAP, DBP, and MAP parameters were lower than the basal level at all times in the group receiving dexmedetomidine, but no clear distinction was seen in the placebo team. These findings were not consistent with those obtained in this study.

HR was significantly higher and different in team C compared to teams A and B at all times. However, the heart rate was significantly decreased in groups A and B at all times compared to T0. No clear distinction was seen between groups A and B. MuniseYildiz et al. [**30**] also showed that MAP and HR were lower at all times compared to the placebo group (p < 0.05). These results were in line with those obtained in our study. Sukhminder Jit Singh Bajwa et al. [**31**] revealed that MAP and HR were significantly lower at all times, of 1, 3, and 5 minutes after the endotracheal intubation in the group receiving dexmedetomidine than in the other group. These results were consistent with those obtained in the present study.

Sameer Arora et al. [**32**] showed that HR, SBP, and DBP were notably reduced in the group receiving dexmedetomidine. These findings were compatible with results of the present research. Ferdi Menda et al. [**29**] showed that HR was clearly less present in the dexmedetomidine team rather than in the placebo team. These results were consistent with the findings in our research.

## Conclusion

It can be excluded that dexmedetomidine significantly and effectively attenuates cardiovascular and hemodynamic responses during laryngoscopy and endotracheal intubation. Dexmedetomidine can reduce SBP, DBP, MAP, and HR without respiratory suppression. When comparing two different doses of the drug, it was shown that 0.5 μg/ kg of dexmedetomidine properly decrease cardiovascular responses. A significant difference was not observed between 0.5 - 1 μg/ kg of dexmedetomidine in reducing HR and MAP.

**Recommendations **

We recommend that a future study should be performed with a large and multi-center sample size, as well as with different doses of dexmedetomidine. Other anesthetic parameters such as depth of anesthesia, and effect of dexmedetomidine on other anesthetic drugs and hemodynamic changes during extubation should also be evaluated. Even the need for analgesic drugs during and after surgery should also be evaluated.

**Limitations**

This was a singular center study with a limited number of cases.

**Acknowledgement**

This was extracted from a general doctoral thesis. Hereby, we acknowledge our gratitude toward all those who helped us in conducting this study including the respected scholars and all officials of Hormozgan University of Medical Sciences and all cases and partners participating in the study and the respectful staff of the operating room of Shahid Mohammadi Hospital in Bandar Abbas.
